# Mercaptonicotinic acid activated thiolated chitosan (MNA-TG-chitosan) to enable peptide oral delivery by opening cell tight junctions and enhancing transepithelial transport

**DOI:** 10.1038/s41598-023-44178-4

**Published:** 2023-10-13

**Authors:** Anubhav Pratap-Singh, Yigong Guo, Alberto Baldelli, Anika Singh

**Affiliations:** 1 Faculty of Land and Food Systems (LFS), University of British Columbia, Vancouver Campus 213-2205 East Mall, Vancouver, BC V6T 1Z4 Canada; 2 Natural Health and Food Products Research Group, Centre for Applied Research and Innovation (CARI), British Columbia Institute of Technology, 4355 Mathissi Pl, Burnaby, BC V5G 4S8 Canada

**Keywords:** Drug delivery, Pharmacology, Diabetes

## Abstract

Recent advances in peptide delivery and nanotechnology has resulted in emergence of several non-parenteral administration routes that replace subcutaneous injections associated with patient discomfort. Thiolated biopolymers are relatively new materials being explored to enhance mucoadhesivity and permeability in these efforts, yet their pH dependent reactivity remains an obstacle. This work focussed on improving the mucoadhesivity of thiolated chitosans by activating them with mercaptonicotinic acid, in a bid to create a novel thiomerized chitosan that can open cell tight junctions for application in oral delivery. The synthesized mercaptonicotinic acid activated thiolated chistoan (MNA-TG-chitosan), along with thiolated chitosan (TG-chitosan) and unmodified chitosan were then used to create insulin nanoparticles (insNPs) using spray drying encapsulation process. Use of MNA-TG-chitosan in place of chitosan resulted in reduction of particle size of insNPs from 318 to 277 nm with no significant changes in polydispersity index (~ 0.2), encapsulation efficiency (~ 99%), insulin loading content (~ 25%) and morphology. Results from in-vitro cytotoxicity on TR146, CaCo2 and HepG2 cell lines revealed no significant effects on cell viability at 50–1000 μg/mL concentration. insNPs encapsulated with the new material, MNA-TG-chitosan, resulted in a 1.5-fold and 4.4-fold higher cellular uptake by HepG2 liver cells where insulin is metabolized, approximately 40% and 600% greater insulin transport through TR146 buccal cell monolayers, and 40% and 150% greater apparent permeability than insNPs encapsulated with unmodified chitosan and TG-chitosan respectively. The higher permeation achieved on using MNA-TG chitosan was attributed to the greater opening of the cell tight junction evidenced by reduction of transepithelial electrical resistance of TR146 buccal cell monolayers. This study demonstrates MNA-TG-chitosan as a promising material for improved peptide oral delivery.

## Introduction

The delivery of peptides and other therapeutic molecules through non-parenteral routes has gained significant attention due to the advancements in peptide delivery and nanotechnology^1,2^. The treatment of diabetes, particularly with insulin, is an area where alternative routes of administration are being explored. Insulin is commonly administered through subcutaneous injections, but these injections present challenges in terms of patient compliance and discomfort^3^. Oral delivery of insulin and other peptide drugs has the potential to provide better glucose homeostasis by more closely mimicking the natural dynamics of these hormones. However, oral delivery faces various obstacles such as low permeation, enzymatic degradation, and low bioavailability^4^. To overcome these challenges, innovative delivery systems have been developed, often utilizing encapsulation of the peptides in nanoparticles, liposomes, and other novel formulations, wherein our group recently proposed the concept for a unidirectional release mucoadhesive buccal tablets for oral delivery of antidiabetic peptide drugs such as insulin, glucagon-like Peptide 1 (GLP-1), and their analogs^5^. Research is actively focused on exploring new engineered materials that can allow peptide delivery through oral or other non-parenteral routes.

Thiomers, which are modified mucoadhesive polymers, have emerged as promising second-generation polymers for enhancing peptide delivery^6^. Thiolated polymers, or thiomers, have been chemically modified to include thiol groups, leading to improved mucoadhesion, enzyme inhibition, permeation enhancement, and efflux pump inhibition. Various thiolated chitosan derivatives have been synthesized by immobilizing thiol groups on the primary amino groups of chitosan^7^. Despite their advantages, the pH-dependent reactivity of thiomers poses limitations to their full potential, as their effectiveness is optimized in specific pH ranges. The reactive form of thiomers is the thiolate anion with pKa is in the range of 8–10, which means that they no longer remain reactive in the pH ranges found in the oral cavity and gastrointestinal canal^6,7^. Although tholated chitosan can open cell tight junctions to allow buccal delivery, they can gets easily oxidized, and its potential cannot be fully utilized. In connection to that, there is an urgent need to design an activated thiolated chitosan and evaluate its suitability for nanoencapsulation of insulin and buccal delivery.

One of the approaches is to explore alternate strategies that can allow development of pH-independent thiomers. Mercaptonicotinic acid (MNA), with a unique structure containing tautomeric thiol (–S–H) and thione (–C=S–) groups that can react both as a nucleophile and a proton donor, has been known to have a pH-independent mechanism of action^8^. This unique structure of mercaptonicotinic acid enables the formation of disulfide bonds (–S–S–) even in the absence of thiolate anions, resulting in a pH-independent action mechanism. Recently a novel thiolated chitosan, called chitosan-6-mercaptonicotinamide (chitosan-6-MNA), has been synthesized by attaching MNA to the amino group (–NH_2_) of chitosan and characterized to have a pH-independent reactivity with good mucoadhesivity and biocompatibility^9^.

This work focuses on addressing the stability issues of thiolated chitosan (TG-chitosan) under neutral and acidic conditions encountered in oral delivery by activating the –S–H groups on TG-chitosan to induce –S–S– linkages to MNA, to create a novel thiomerized chitosan that can open cell tight junctions for non-parenteral delivery. Subsequently, the synthesized MNA-TG-chitosan was used to encapsulate insulin nanoparticles (insNPs) through a spray drying process. Earlier, Guo et al.^10^ demonstrated the production of insNPs using spray drying technique, as opposed to conventional freeze drying technique, to be used in the buccal tablets proposed by Pratap-Singh et al.^5^. In this study, the performance of MNA-TG-chitosan insNPs was compared with insNPs encapsulated with unmodified chitosan and TG-chitosan in terms of particle size, polydispersity index, encapsulation efficiency, insulin loading content, and morphology of the insNPs. Furthermore, in vitro cytotoxicity studies were conducted on different cell lines (intestinal, liver and buccal cells) and to assess the biocompatibility of MNA-TG-chitosan. The effectiveness of MNA-TG-chitosan in increasing cellular uptake of insulin on hepG2 cells and insulin transport, apparent permeability and tight junction opening of TR146 buccal monolayers was assessed and compared with TG-chitosan encapsulated and unmodified chitosan encapsulated insNPs and free insulin.

## Results and discussion

### Synthesis and characterisation of TG-chitosan and MNA-TG-chitosan

**Figure 1:**
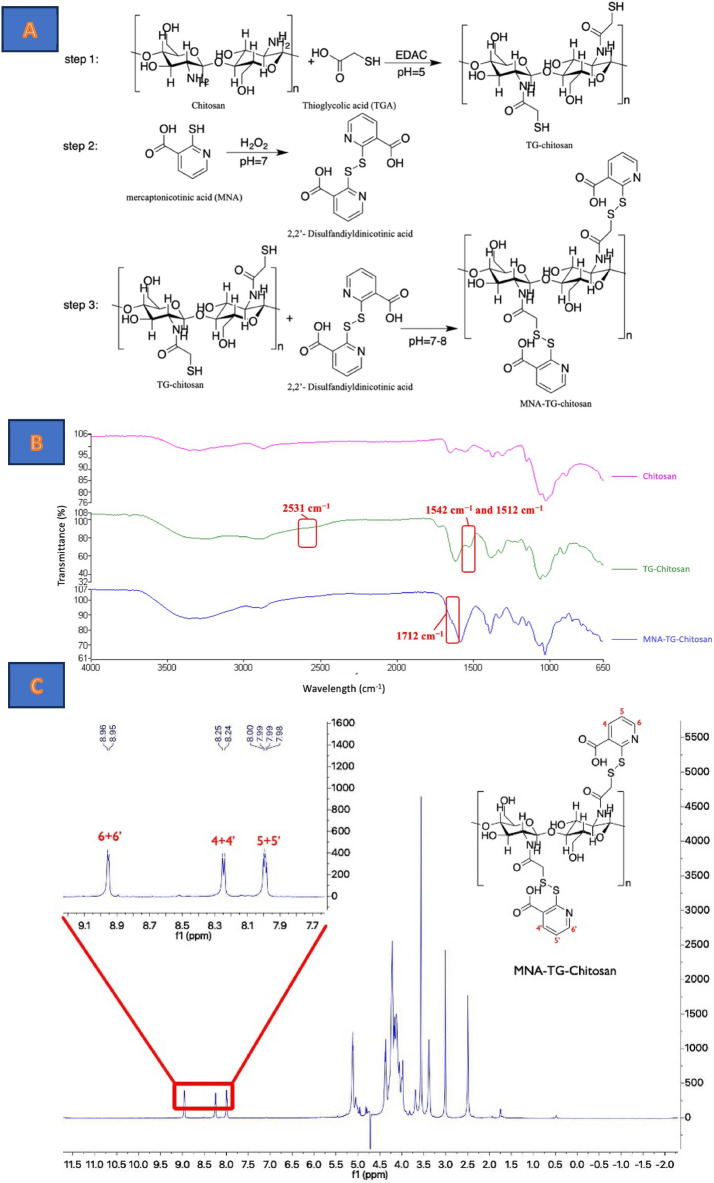
(**A**) Synthesis routes of TG-Chitosan and MNA-TG-Chitosan, (**B**) ATR-FTIR of unmodified chitosan, TG-chitosan and MNA-TG-chitosan, and (**C**) NMR-characterization of MNA-TG-chitosan to confirm MNA-TG-chitosan synthesis.

MNA-TG-chitosan was synthesized was from chitosan, thioglycolic acid and MNA using a 3-step process comprised of (a) production of TG-chitosan by thiolation of chitosan by thioglycolic acid at pH 5 in presence of cross-linking agents like 1-ethyl-3-(3-dimethylamino-propyl) carbodiimide hydrochloride (EDAC) etc. in Step 1; (b) dimerization of MNA in presence of hydrogen peroxide at pH 7 to produce disulfandiyldinicotinic acid in Step 2; and (c) production of MNA-TG-chitosan by activating the TG-chitosan with disulfandiyldinicotinic acid at pH 7–8 in Step 3. The synthesis chemistry is highlted in Fig. 1A.

In the presence of thioglycolic acid, thiol groups were bound to chitosan via a mediation between the carboxylic group of thioglycolic acid and primary amino groups of chitosan. The immobilization of thiol groups from chitosan was accomplished by exploiting the reactivity of the primary amino group at the 2 position of the glucosamine subunit. It has been shown in previous studies^8,9^ that a degree of modification of 25–250 μmol thiol groups per gram of chitosan results in the highest improvement in the mucoadhesion and permeation-enhancement properties. TG-chitosan resulted in a thiol group content of 218 ± 33 μmol per gram of the polymer.

The thiol groups on TG-chitosan were then activated with MNA dimers to create disulfide linkages between TG-chitosan and MNA dimers, as shown in Fig. 1A. MNA-TG chitosan was found to contain 138 ± 21 μmol of disulfide bond per gram of polymer. This indicates more than 63% of the thiol groups have been activated, consistent with the previous result based on activated MNA-TG-cellulose^11^ and MNA-chitosan^9^.

Attenuated total reflectance Fourier transform infrared spectroscopy (ATR-FTIR) showed the absorption peaks corresponding to the characteristic amide I band (1665 and 1622 cm^−1^), amide II bands (CN stretching and NH bending at 1542 cm^−1^ and 1512 cm^−1^), NH stretch (3303 cm^−1^ and 3262 cm^−1^), and a weak peak (at 2531 cm^−1^) indicating SH conjugation for both chitosan and TG-chitosan (Fig. 1B)^12^. MNA-TG-chitosan showed NH bends (at 1653–1619 cm^−1^) and presence of amide bonds in contrast to the unmodified chitosan in the 1712 cm^−1^^11^. The nuclear magnetic resonance (NMR) data (Fig. 1C) detected the coupling reagent disulfandiyldinicotinic acid which confrmed that the synthesis of MNA-TG-chitosan was successfully conducted in this study.

### Effect of using MNA-TG-chitosan and TG-chitosan as a coating material for encapsulated insNPs preparation

As this is the first time MNA-TG-chitosan has been synthesized, its’ potential to replace chitosan as a coating material for the encapsulated insNPs optimized by Guo et al.^10^ was investigated. The optimized insNPs with chitosan resulted in 318 nm of mean particle size, 0.18 of polydispersity index (PDI), 99.03% of encapsulation efficiency (EE), 9.8 mv of zeta potential, and 25.19% (m/m) of insulin loading content (Table 1). On using TG-chitosan and MNA-TG chitosan, as shown in Table 1, the mean particle sizes of dehydrated particles decreased to 235 and 277 nm respectively, with no significant changes in PDI, encapsulation efficiency and insulin loading content. The mean particle size of optimized insNPs encapsulated with TG-chitosan decreased more than insNPs encapsulated with MNA-TG-chitosan with no significant change in PDI, EE, and loading content (Table 1). This could be attributed to the larger size of MNA-TG-chitosan than TG-chitosan due to the presence of MNA ring on TG-chitosan. This larger side-chain, in turn allowed greater cross-linking improving the encapsulation efficiency of MNA-TG-chitosan insNPs when compared to TG-chitosan insNPs.

**Table 1:** Properties of freshly prepared and reconstituted insNPs prepared with unmodified chitosan, TG-chitosan and MNA-TG-chitosan.

		Chitosan insNPs	TG-chitosan insNPs	MNA-TG-chitosan insNPs
Z-average diameter (nm)	Dry	318 ± 18^c^	235 ± 16^a^	277 ± 14^b^
	Reconstituted	457 ± 34^e^	323 ± 29^c^	389 ± 33^d^
Polydispersity index	Dry	0.18 ± 0.01^a^	0.13 ± 0.03^b^	0.21 ± 0.02^a^
	Reconstituted	0.21 ± 0.04^a^	0.19 ± 0.05^a^	0.21 ± 0.03^a^
Encapsulation efficiency (%)	Dry	99.03 ± 0.33^a^	98.43 ± 0.64^a^	98.79 ± 0.43^a^
	Reconstituted	98.09 ± 0.21^a^	98.31 ± 0.39^a^	98.43 ± 0.53^a^
Loading content (%)	Dry	25.19 ± 0.37^a^	25.35 ± 0.53^a^	25.03 ± 0.43^a^
	Reconstituted	24.87 ± 0.43^a^	25.02 ± 0.26^a^	24.92 ± 0.52^a^

^a-e^ Means with same letters as superscripts are not significantly different from each other for each property studied.

**Figure 2:**
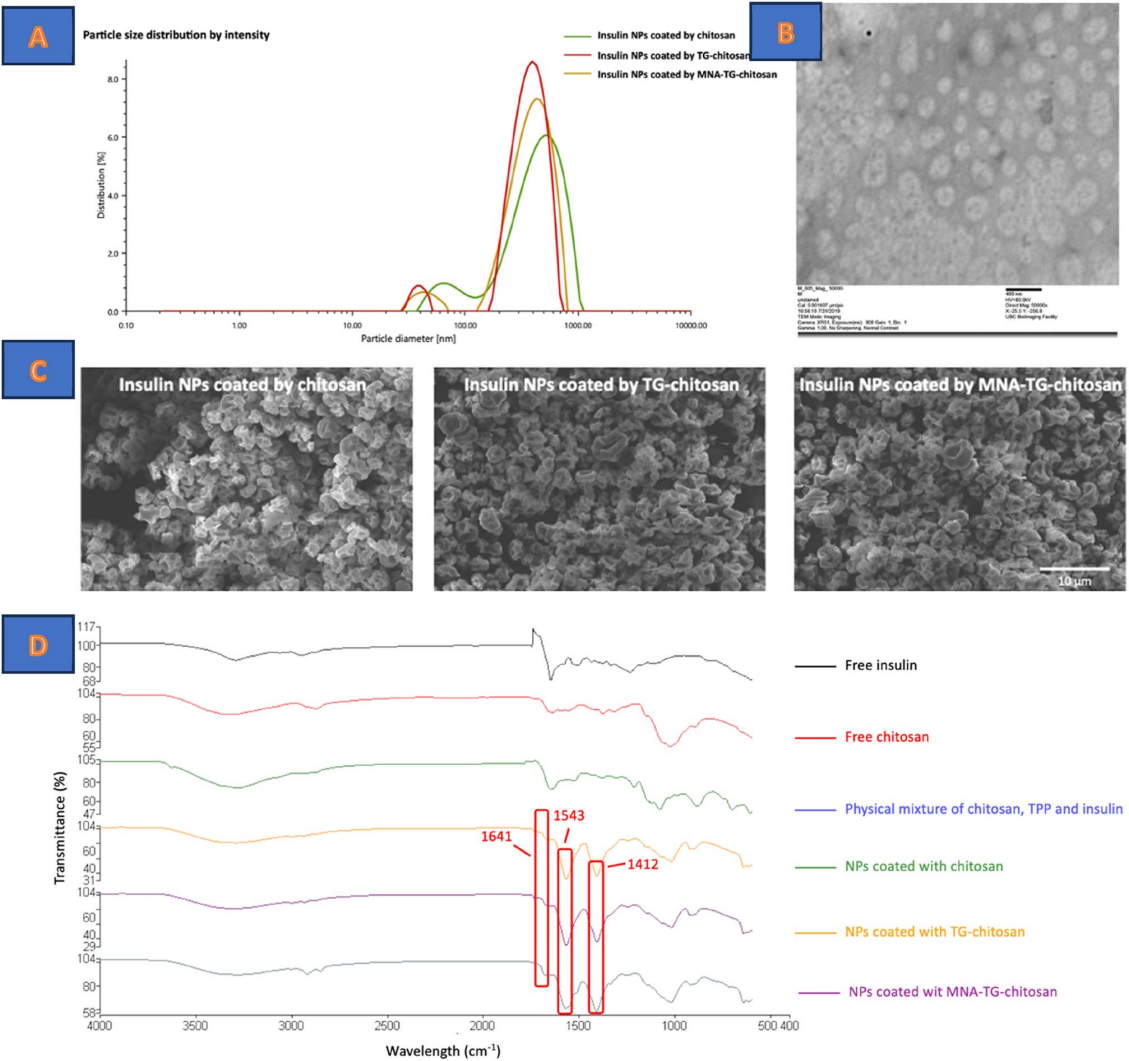
Characterization of insulin nanoparticles coated with different chitosans (**A**) size distribution of insulin nanoparticles coated with chitosan, TG-chitosan and MNA-TG-chitosan after reconstitution; (**B**) TEM micrographs of the optimized insulin nanoparticles; (**C**) SEM image of spray dried insulin insulin coated with chitosan, TG-chitosan and MNA-TG-chitosan (**D**) FTIR-ATR spectra of free insulin, chitosan, physical mixture of chitosan/TPP/insulin and insulin nanoparticles.

After reconstitution, the mean particle size of the insNPs encapsulated with chitosan was increased to 457 nm, while the PDI, EE, and loading content did not significantly change (p < 0.05) (Fig. 2A). The reconstitution ability of the dehydrated insNPs encapsulated with TG-chitosan and MNA-TG-chitosan showed smaller particle sizes than the insNPs encapsulated with chitosan (Table 1).

Based on the transmission electron microscope (TEM) results, the optimized insNPs were spherical and discrete with relatively uniform size (Fig. 2B). Scanning electron microscopy (SEM) results suggested the spherical structure with a wrinkled surface after spray drying without any bulking agents (Fig. 2C). The morphology of the dehydrated insNPs encapsulated with TG-chitosan and MNA-TG-chitosan had no visible changes (Fig. 2C).

Free insulin, chitosan, physical mixture of chitosan, sodium tripolyphosphate (TPP), insulin and all insNPs were characterized by using FTIR-ATR spectroscopy. Noticeably, increases in the band intensities at 1641, 1543 and 1412 cm^−1^ were observed in encapsulated insNPs (Fig. 2D). These increases in intensities are associated with crosslinking among chitosan, TPP and insulin.

All these results indicate that the TG-chitosan and MNA-TG-chitosan improved the spray drying characteristics insNPs, initially optimized for chitosan by Guo et al.^10^. Moreover, a previous study proved that the TG-chitosan could significantly decrease the mean particle sizes of nanoparticles because of the formation of disulfide (–S–S–) bonds in or outside the nanoparticles^13,14^, which was consistent with our results. However, in our case, most of the thiol (–S–H) groups of MNA-TG-chitosan have been connected to the MNA group (Fig. 1). Therefore, only part of them can form disulfide linkages and thus MNA-TG-chitosan’s capacity to decrease mean particle size was lower than TG-chitosan.

### In-vitro release behavior of insNPs

**Figure 3:**
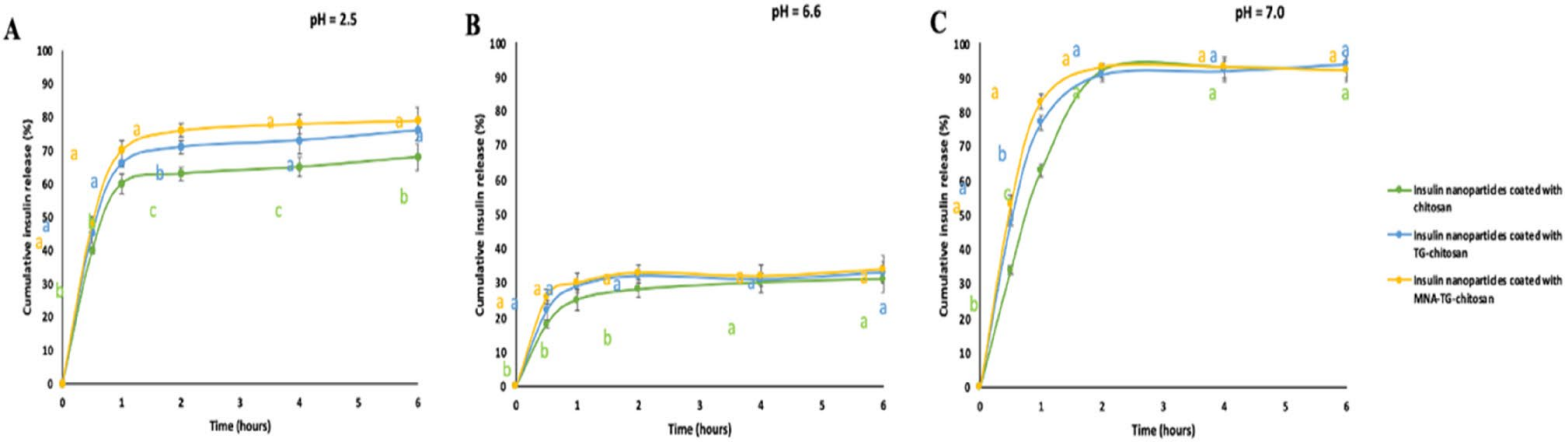
Release behaviors of insulin nanoparticles: (**A**) Release behaviors of insulin nanoparticles in pH = 2.5 solution; (**B**) Release behaviors of insulin nanoparticles in pH = 6.6 solution; (**C**) Release behaviors of insulin nanoparticles in pH = 7.0 solution. The significance of the differences among different samples at the same time point was analyzed by one-way analysis of variance (ANOVA). Superscript with different alphabets are significantly different (p < 0.05).

The release profile of insulin from chitosan, TG-chitosan and MNA-TG-chitosan insNPs is shown in Fig. 3. The total cumulative insulin release from chitosan, TG-chitosan and MNA-TG-chitosan nanoparticles were more than 60%, 25% and 90% of insulin in first 2 h in pH 2.5, 6.6 and 7, respectively for all cases. Insulin release was partly controlled by the porous network of nanoparticles. It was obvious that all formulations exhibited a burst release of insulin in the first half hour, and then a constant controlled release, as observed earlier^10^. The initial burst release of insulin was caused by insulin attached to the surface or loosely associated with the nanoparticles. Insulin was released from the nanoparticles rapidly at the beginning due to the large concentration gradient between the release medium and the polymer surface. Furthermore, insulin dispersed within the nanoparticles could have escaped through pores created during the dehydration process, thus causing initial release. Following the initial burst release, the insulin/TPP/chitosan complexation led to a prolonged release period.

A previous study found that disulphide bonds result in higher cohesive properties^8^. Often, a controlled release system must maintain its cohesion and stability over the period of the drug release. It was noticed from the results that the MNA-TG-chitosan insNPs released insulin fastest followed by TG-chitosan insNPs and unmodified chitosan insNPs (Fig. 3), although the cumulative release was not significantly different. This is due to the disulphide bonds (–S–S–) formation in TG-chitosan and MNA-TG-chitosan limiting the insulin/TPP/chitosan complexation, since less chitosan amino groups (–NH_2_) were available for interaction.

### In-vitro cellular uptake on HepG2 cells

The liver is the primary organ where insulin performs its physiological function. HepG2 cell is a human liver cancer cell line commonly utilized as a hepatocyte absorption model in vitro. Herein, HepG2 cells, a human liver cancer cell line as a hepatocyte absorption model in vitro, was used to evaluate the cellular uptake of free insulin, and insNPs coated with chitosan, TG-chitosan and MNA-TG-chitosan.

**Figure 4:**
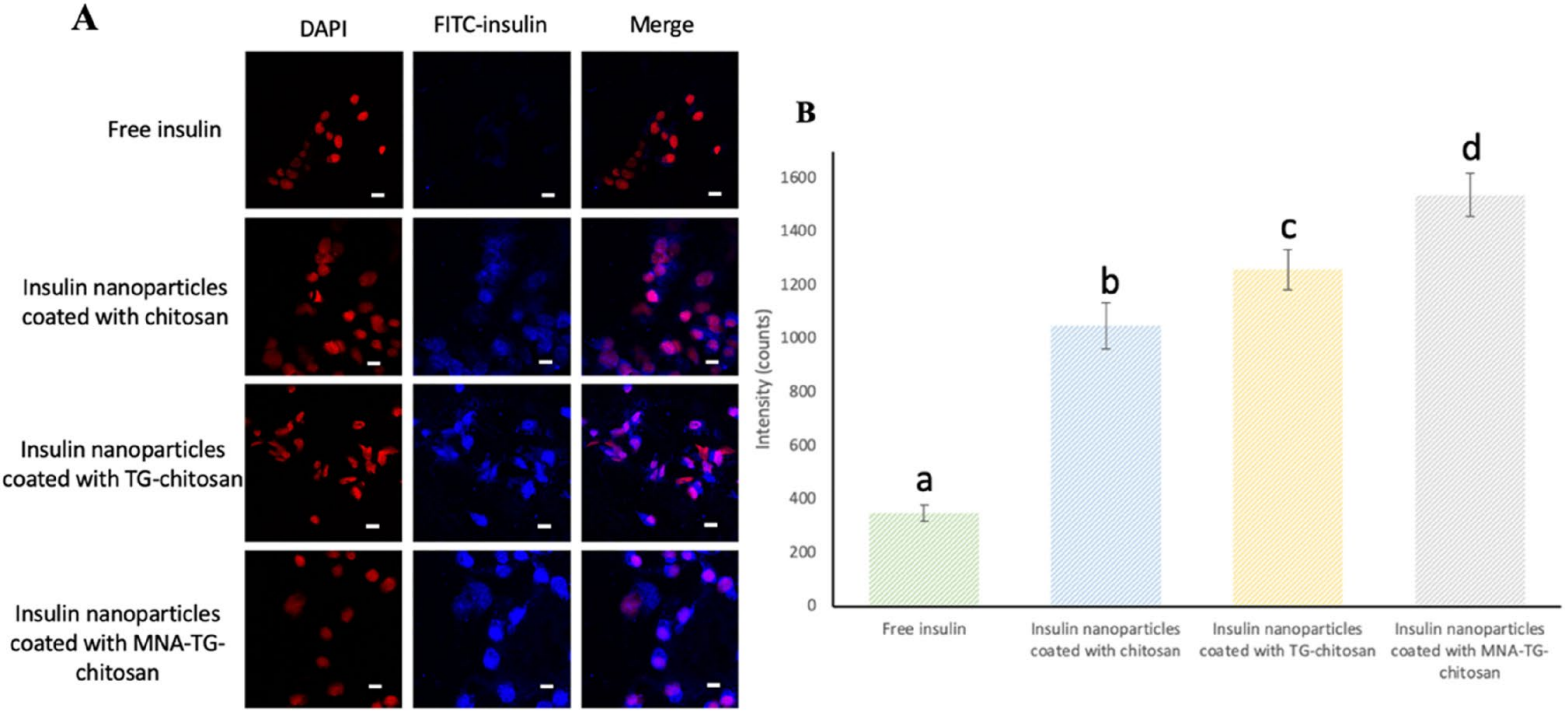
HepG2 cellular uptakes after 4 h incubation with free insulin and insulin nanoparticles: (**A**) Distribution of FITC-insulin uptaken by HepG2 cells (white bar = 10 μm). (**B**) Geometric mean values of the fluorescence intensities of the flow cytometry analysis. The significance of the differences among different samples at the same time point was analyzed by one-way analysis of variance (ANOVA). Superscript with different alphabets are significantly different (p < 0.05).

The cellular uptakes were quantified using flow cytometry and visually by confocal laser scanning microscopy (CLSM) observation, wherein the intracellular fluorescence intensities (Fig. 4A) of redissolved spray-dried insNPs encapsulated with chitosan, TG-chitosan and MNA-TG-chitosan were 2.9, 3.6, and 4.4-fold higher than the intensity of the free FITC-insulin group, respectively (Fig. 4B). Accordingly, these results showed a higher cellular uptake when MNA-TG-chitosan encapsulated the insulin, as compared to TG-chitosan and chitosan.

Besides the smaller particle sizes of TG-chitosan and MNA-TG-chitosan insNPs, the cell uptake mechanism is also controlled by reactions between functional groups on the surface of the insNPs with exofacial thiols of transmembrane proteins^15^. It can be done by exchanging disulfide groups on the surface of the nanoparticles with thiol groups on the transmembrane proteins so that a new disulfide bond can be formed between the nanoparticles and the membrane protein, resulting in increased insulin transport^16^. In our case, as MNA-TG-chitosan had more –S–S– groups, it displayed greater mucoadhesivity and could bind more tightly to the cell transmembrane proteins to increase the cellular uptake of insulin.

### In-vitro insulin transport through the TR146 buccal monolayers and cell tight junction tests

**Figure 5:**
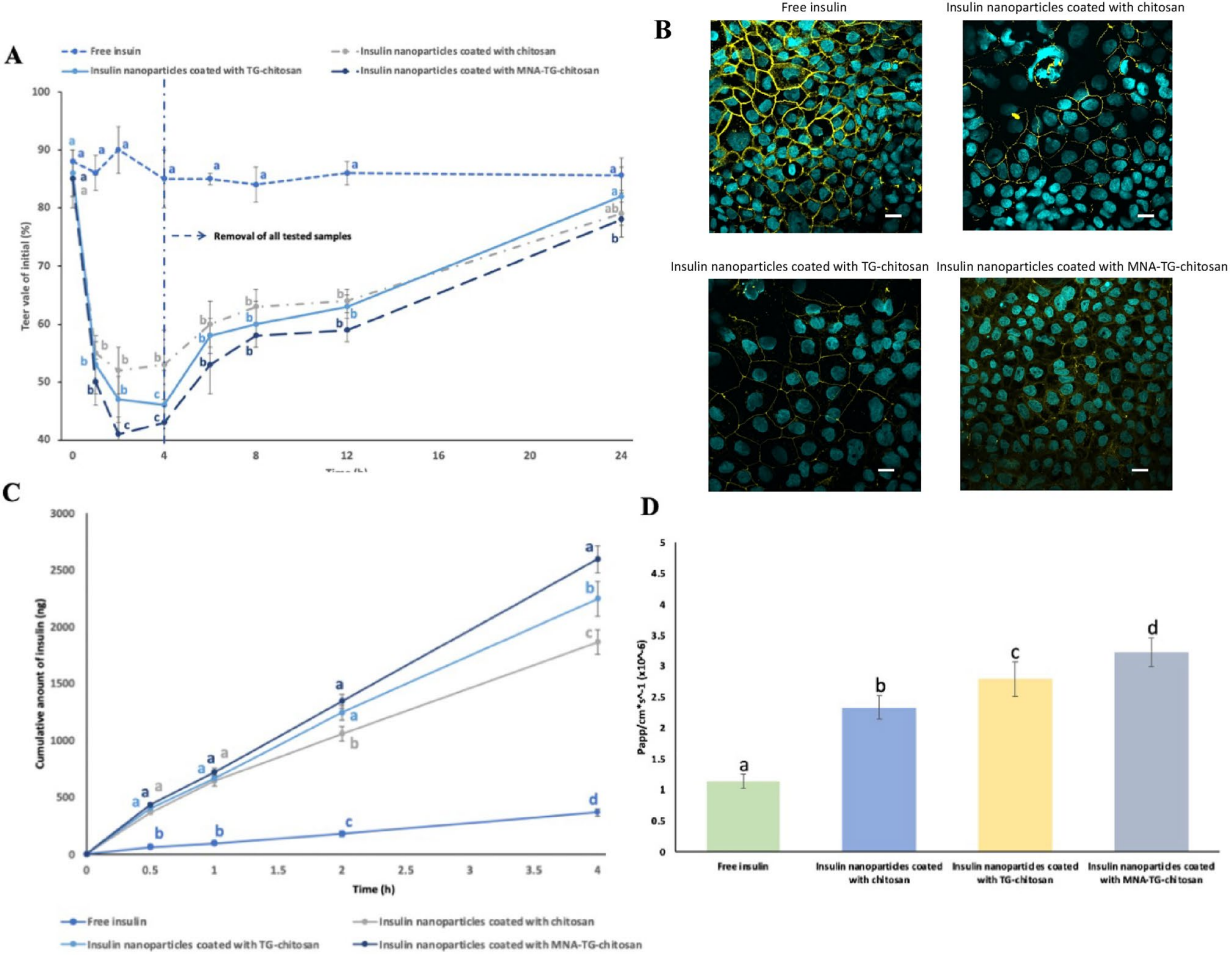
Effect of chitosan, TG-chitosan and MNA-TG-chitosan insNPs on TR146 monolayers: (**A**) Effects of TEER values; (**B**) Fluorescence images of TR-146 monolayer stained for tight junction protein ZO-1 after incubation with insNPs (white bar = 20 μm); (**C**) Effect on transepithelial insulin transport across TR-146 monolayer; (**D**) P_app_ values of insNPs across the TR-146 cell monolayer. The significance of the differences among different samples at the same time point was analyzed by one-way analysis of variance (ANOVA). Superscript with different alphabets are significantly different (p < 0.05).

The impact of MNA-TG-chitosan was also assessed on transepithelial transport of insulin from insNPs through TR146 buccal cell monolayers to establish if the newly synthesized material could be used for buccal delivery of insulin as proposed by Pratap-Singh et al.^5^. insNPs encapsulated with chitosan, TG-chitosan, and MNA-TG-chitosan significantly reduced the transepithelial electrical resistance (TEER) value of TR146 cells compared to untreated cells and free cells insulin-treated cells (Fig. 5A), suggesting that chitosan-based insNPs are effective in enhancing drug permeation across epithelium principally by temporarily opening tight junctions that connect epithelial cells^17^ based on interaction with thiol groups of membrane-bound enzymes and proteins according to different mechanisms^18^. Also, it can be observed that the MNA-TG-chitosan induced the highest reduction of the TEER value, followed by TG-chitosan and chitosan. Further, after the test samples were removed and the medium was refilled, a gradual recovery in TEER values was observed, suggesting that opening the tight junctions was a transient and reversible process.

Direct interactions between chitosan molecules and integrin receptors on cell membranes play a critical role in the chitosan-mediated opening of tight junctions. As a result, the conformation of integrin receptor was altered, resulting in clustering along the cell border, causing F-actin reorganization, FAK and Src phosphorylation, and ZO-1 downregulation, which led to disruption of the tight junction. Moreover, as shown in Fig. 5B, the band of ZO-1 in the group treated with free insulin appeared continuously between adjacent cells. After incubation with insNPs encapsulated with chitosan, TG-chitosan, and MNA-TG-chitosan, the ZO-1 staining bands were segmented and discontinuous, indicating the opening of tight cell junction. Besides, the signals of ZO-1 staining were weakest for the insNPs with MNA-TG-chitosan followed by TG-chitosan and chitosan, which was consistent with the results of TEER values.

The cumulative amount of insulin transported within 4 h through the TR-146 monolayer (Fig. 5C) resulted in the greatest amount of insulin transported for insNPs encapsulated with MNA-TG-chitosan group (2593 ± 121 ng), followed by TG-chitosan group (2246 ± 154 ng), followed by chitosan (1863 ± 109 ng), and lowest for free insulin (368 ± 32).

To compare the efficiency of insulin transport, P_app_ values or absolute quantity of nanoparticles are more appropriate than cumulative amounts of insulin, since these measurements are extremely dependent on the experimental setup, such as the concentration of nanoparticles loaded and incubation time. The apparent permeability (P_app_) value of insulin followed a similar trend with insNPs with MNA-TG-chitosan (3.22 ± 0.23 × 10–6 cm/s) being highest among all tested groups followed by TG-chitosan (2.79 ± 0.28 × 10–6 cm/s), chitosan (2.33 ± 0.19 × 10–6 cm/s) and free insulin (1.14 ± 0.11 × 10–6 cm/s). which is more appropriate than cumulative amounts of insulin to compare insulin transport efficiency (Fig. 5D).

Besides the fact that the TG-chitosan and MNA-TG-chitosan can open the cell tight junction, they were also proved to have the efflux pump inhibitory properties. By forming disulfides with cysteine-substructures of natural proteins located within the channel of efflux pumps, they are capable of reversibly inhibiting efflux pumps and thus increase the cell penetration. The reason TG-chitosan and MNA-TG-chitosan can open the tight junction more widely than chitosan is based on interaction with thiol groups of membrane bound enzymes and proteins according to different mechanism. Thiomers are, for example, capable of inhibiting protein tyrosine phosphatase through glutathione. The inhibition of this enzyme inhibits the dephosphorylation of the tyrosine subunits on occludin, thereby resulting in the opening of the tight junction. In our case, the disulfide groups on the surface of the MNA-TG-chitosan made it more mucoadhesive as well as imparted the capability of opening the cell tight junction wider compared with TG-chitosan, resulting in better transepithelial insulin transport through TR146 buccal cell monolayers and insulin cellular uptake by hepG2 liver cell lines.

### In-vitro cytoxicity of insNPs on TR146, HepG2 and Caco2 cell lines

**Figure 6:**
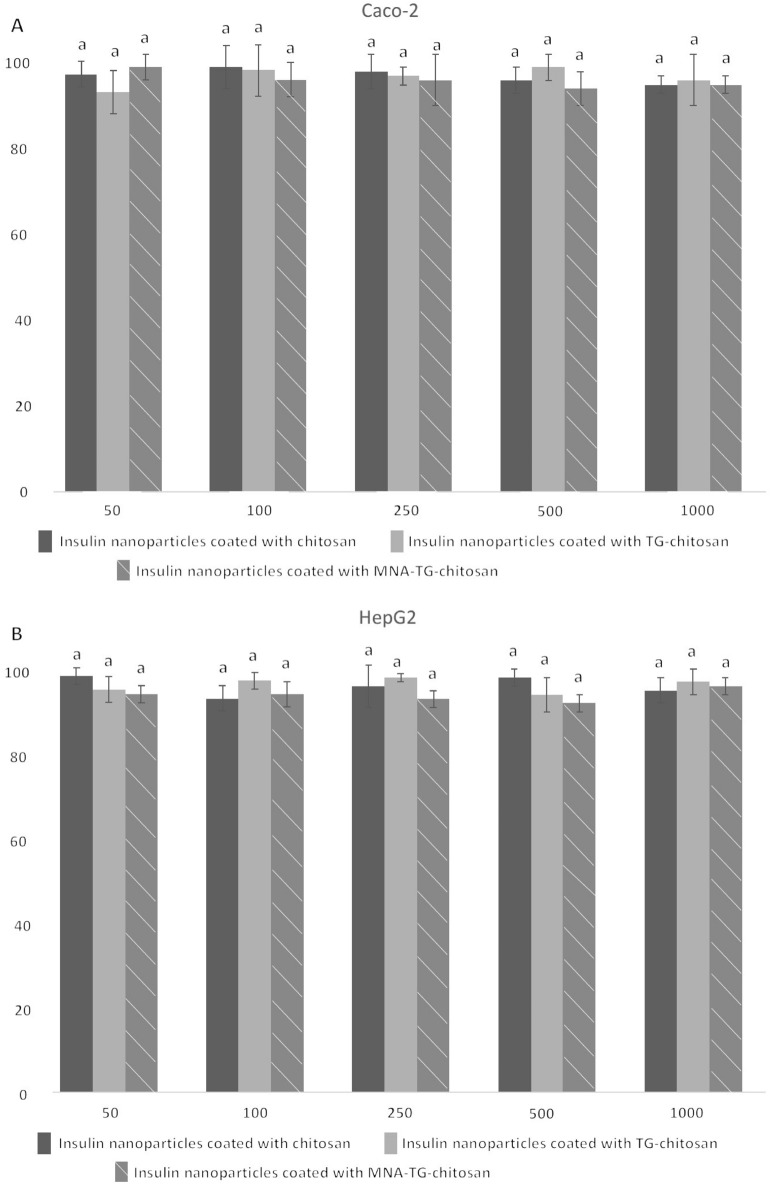

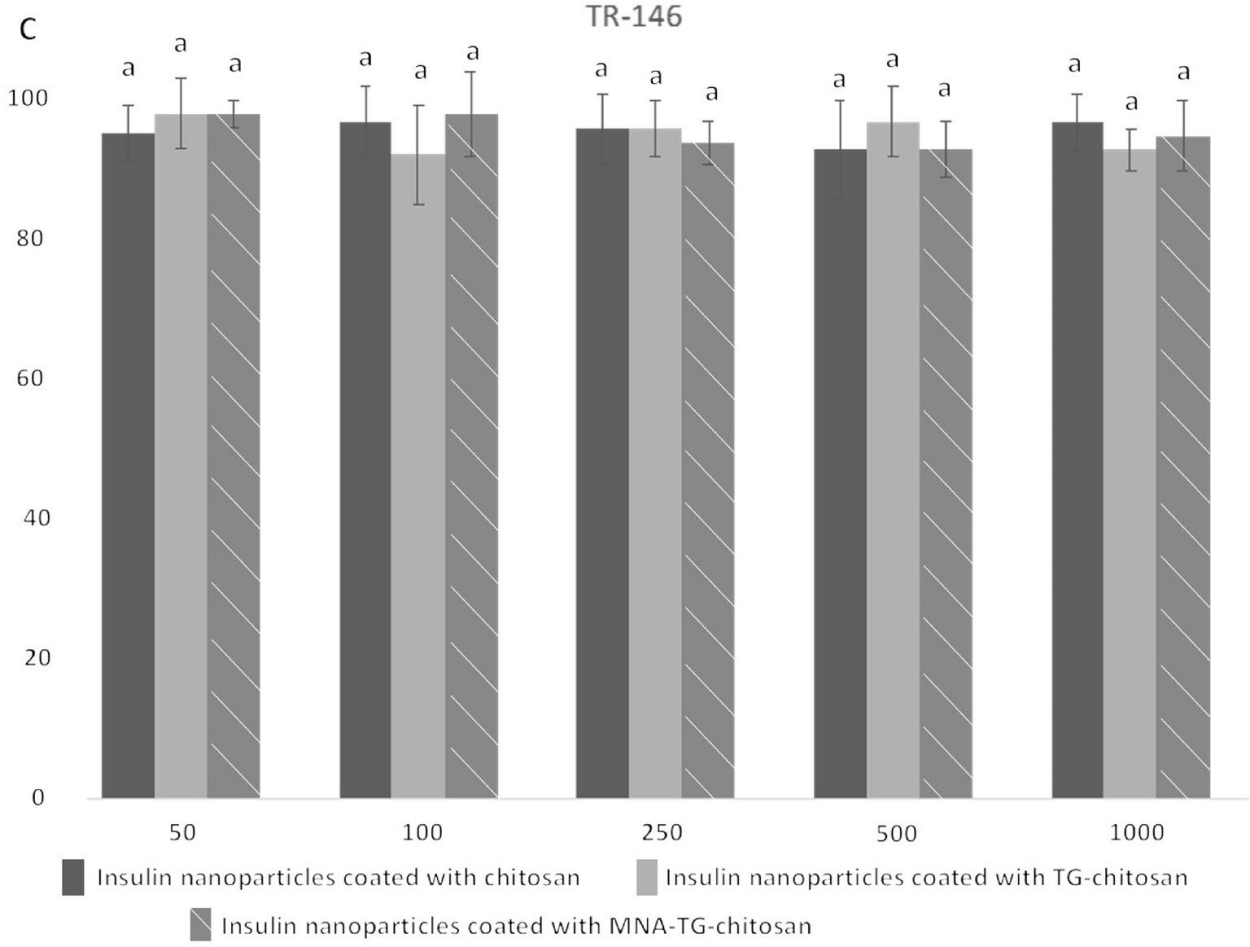
Cytotoxicity of the insulin particles encapsulated with chitosan, TG-chitosan and MNA-TG-chitosan in (**A**) Caco-2; (**B**) HepG2; and (**C**) TR-146 cell lines show no significant differences.

As this is the first study using MNA-TG-chitosan as part of insNPs used to deliver insulin to humans, the impacts of the insNPs were tested all possible human cells insNPs might contact in human body. As buccal and gastrointestinal delivery candidates, buccal cells (TR-146) and intestinal cells (CaCo2) were tested, and as the liver is the primary organ where insulin performs its physiological function^19^, liver cells (HepG2) were also used in this test. All insNPs encapsulated with chitosan, TG-chitosan, and MNA-TG-chitosan were found to have no significant impact on the cell viability in the 3-(4,5-Dimethylthiazol-2-yl)-2,5-diphenyltetrazolium bromide (MTT) test at the concentration of 50–1000 μg/mL (Fig. 6), which indicated that all insNPs could be safely used to reach the therapeutic window. This proves that MNA-TG-chitosan was toxically safe and similar to TG-chitosan and chitosan, both regulatorily approved as candidates for drug delivery.

## Materials and methods

### Materials

Chitosan (Average Mw 100 KDa, 75–85% deacetylated), Carbopol 934P (viscosity 800–11,000 cP), hydroxypropyl methylcellulose (viscosity 2600–5600 cP), and ethylcellulose (viscosity 10 cP) were purchased from Sigma-Aldrich. (Oakville, Ontario, Canada). Sodium tripolyphosphate (TPP) and sodium alginate (low viscosity) was purchased from VWR (Radnor, Pennsylvania, USA). Recombinant human insulin was obtained from Fisher Scientific (Waltham, Massachusetts, USA). Fluorescein isothiocyanate (FITC)-labelled human insulin and the 4′,6-Diamidino-2-phenylindole dihydrochloride (DAPI) were obtained from Sigma-Aldrich. (Oakville, Ontario, Canada). The HepG2 and Caco-2 cell line was obtained from ATCC (Manassas, Virginia, USA) while TR-146 Cell line was purchased from Sigma-Aldrich. (Oakville, Ontario, Canada). Other reagents were of analytical or chromatography grade.

### Cell culture protocols

HepG2 cells, human hepatocellular carcinoma cell line, were cultivated in Nunc™ cell culture petri dishes (Thermo Fisher, NY, USA) with 60 mm diameter using Dulbecco's Modified Eagle Medium (DMEM) containing 10% of fetal calf serum, 100 IU/mL of penicillin and 100 μg/mL of streptomycin. The culture was kept at the environment of 37 °C, 95% relative humidity with 5% CO_2_. Media were changed every 2–3 days depending on the growth rate. Cells were seeded at a concentration of 2 × 104 cells/cm2 for subcultivation twice a week using 0.25% trypsin–EDTA^10^.

Caco 2 cells, widely used as a model of the intestinal epithelial barrier, were cultivated in Nunc™ cell culture petri dishes (Thermo Fisher, NY, USA) with 60 mm diameter using Dulbecco’s Modified Eagle Medium (DMEM) containing 10% of fetal calf serum, 100 IU/mL of penicillin and 100 μg/mL of streptomycin. The culture was kept at the environment of 37 °C, 95% relative humidity with 5% CO_2_. Media were changed every 2–3 days depending on the growth rate. Cells were seeded at a concentration of 2 × 104 cells/cm2 for subcultivation once a week using 0.25% trypsin–EDTA.

TR146 cells, appropriate for drug transport studies and mimic normal human buccal epithelium, were cultivated in Nunc™ cell culture petri dishes (Thermo Fisher, NY, USA) with 60 mm diameter using Nutrient Mixture F-12 Ham containing 10% of fetal calf serum, 100 IU/mL of penicillin and 100 μg/mL of streptomycin. The culture was kept at the environment of 37 °C, 95% relative humidity with 5% CO_2_. Media were changed every 2–3 days depending on the growth rate. Cells were seeded at a concentration of 2 × 104 cells/cm^2^ for subcultivation once a week using 0.25% trypsin–EDTA (10).

### Synthesis, purification and characterization of MNA-TG-chitosan and TG-chitosan

Thiolated chitosan (TG-chitosan) was synthesized using a method previously described ealier^12^, from which MNA activated TG-chitosan (MNA-TG-chitosan) was synthesized. The synthesis route is shown in Fig. 1A. The MNA-TG-chitosan and TG-chitosan were characterized for thiolated groups and sulphide bonds using Ellman's assay, Fourier Transform Infrared Red (FTIR) spectroscopy and nuclear magnetic resonance (NMR).

*TG chitosan synthesis* Briefly, 1 g chitosan was first dissolved in 100 mL HCl with the concentration of 0.1 M. Then, 1 mL of thioglycolic acid (TGA) was added and the pH was adjusted to 5.0 with NaOH. EDAC of 50 mM was added to activate the carboxylic moiety of TGA under continuous stirring for four hours at room temperature. The resultant TG-chitosan solution was dialyzed for 8 h against 5L of 5 mmol/L HCl with 1% of NaCl (twice in the dark), then 8 h against 5 L of 5 mmol/L HCl (twice in the dark) to avoid interactions between cationic chitosan and anionic sulfhydryl ligand. Purified TG-chitosan were then freeze dryed and stored at 4 °C in dark until further use.

*MNA-TG-chitosan synthesis* A total of 2 g of mercaptonicotinic acid (MNA) were hydrated in 50 mL deionized water while the pH of the solution was adjusted to 7. Subsequently, 5.3 mL of 30% hydrogen peroxide was added to the solution dropwise, maintaining the pH at 7. Next, the pH of the solution was adjusted to 2.5 by using 0.5 M HCl, followed by filtering and washing the 2-2-disulfandiylnicotinic acid precipitate. After washing, the 2-2-disulfandiylnicotinic acid precipitate was dried in a vacuum dehydrator and stored at 4 °C, pending further use. Preactivation was carried out by reconstituted 2-2-disulfandiylnicotinic acid solution to freshly prepared TG-chitosan solution before spray drying in the ratio of 1:2. Using 5 M NaOH, the pH of the reaction mixture was set to 7.5 when it was stirred continuously for six hours at room temperature. Following the reaction, the MNA-TG-chitosan was purified using dialysis procedure in 5 L of demineralized water at 10 °C for 8 h in the dark for three times^9^. Purified MNA-TG-chitosan were then freeze dryed and stored at 4 °C in dark until further use.

*Thiol groups* For thiol groups determination, briefly, MNA-TG-chitosan and TG-chitosan were mixed with phosphate buffer and incubated at room temperature for 30 min. Then, Ellman’s reagent was added and incubated at room temperature for 90 min in dark. After 90 min, aliquots of 100 μL were transferred to a 96-well plate and the absorbance was measured at 450 nm by Tecan infinite M200 pro spectrophotometer plate reader (Tecan, Männedorf, Switzerland).

*Disulfide bonds* For disulfide bonds determination, MNA-TG-chitosan and TG-chitosan were dissolved in 50 mM Tris buffer. Then, 4% sodium borohydride solution was added and incubated at 37 °C for 60 min. After the incubation, HCl was added followed by Ellman’s reagent and further incubated for 90 min at room temperature in dark.

*ATR-FTIR* The FTIR of MNA-TG-chitosan, TG-chitosan and chitosan were conducted by Spectrum 100 FTIR spectrophotometer (PerkinElmer, Waltham, Massachusetts, USA) equipped with universal ATR sampling accessories (PerkinElmer, Waltham, Massachusetts, USA). Signal averages were obtained from 16 scans in the frequency range of 4000–600 cm^2^ at a resolution of 4 cm^2^.

*NMR* Furthermore, 1H-NMR spectra were taken on a Bruker Avance Cryoprobe 600 MHz spectrometer (Bruker, Billerica, Massachusetts, USA). The data was acquired and processed with MestReNova Ver. 12.0.4 (Mestrelab Research, Santiago de Compostela, Spain). 1H NMR spectra were got by Bruker pulse programs with standard acquisition parameters at 298 K (25 °C).

### insNPs preparation & characterization

insNPs were prepared using the optimized method from our previous research^10^. Briefly, 1 mg/mL of chitosan, TG-chitosan or MNA-TG-chitosan was dissolved in 0.1% acetic acid and then mixed with insulin and TPP at a ratio of 0.5:1:2.5 (TPP:insulin:chitosan) under 10,000 rpm stirring by polytron PCU-2-110 high-speed homogenizer (Brinkmann Ind. Westbury, NY, USA). After adjusting pH to 6.1, the mixed solution was maintained under high-speed stirring for another 30 min to facilitate chitosan-TPP cross-linking. Although, with respect to chitosan protonation, a lower pH than 6.1 may be preferrable, for buccal or sublingual delivery insNPs, a pH closer to neutral pH is desirable. Our previous study^10^ revealed that the particle size of the insNPs formed after spray drying encapsulation showed a continuous decrease before the pH reached the value of 6.1, and a significant increase in size was observed at pH > 6.1. This is because as pH rises, insulin molecules gain a negative surface charge; thus, favoring electrostatic interactions with chitosan/ Sodium tripolyphosphate (TPP) complex and resulting in small particle sizes and high EE. However, when pH was tuned to 6.5, deprotonation of amino groups on chitosan occurred, leading to the folding of chitosan.

To further homogenize and decrease the particle sizes of the insNPs, they were ultrasonicated for another 30 min using a probe type ultrasonicator (UP 200ST, Hielscher Ultrasonics, Teltow, Germany). insNPs were dehydrated using a Buchi mini spray dryer B-290 (BÜCHI, Flawil, Switzerland) with an inlet temperature of 100 °C and outlet temperatures varying between 80 and 85 °C, f feeding flow of 3 L/min, and airflow of 4 L/min at 90 °C. The synthesized insNPs were characterized for z-average diameter, polydispersity index (PDI), zeta potential, morphology, encapsulation efficiency, loading content, reconstitution capacity and in-vitro release behavior.

*Particle size and zeta potential* The Z-average diameter, polydispersity index (PDI) and zeta potential of all insNPs prepared were tested using dynamic light scattering (DLS) measurements using Litesizer 500 (Anton Paar, Graz, Austria).

*Morphology* Morphology was characterized by Hitachi H7600 Transmission electron microscopy (TEM) (Hitachi, Tokyo, Japan), and dry insNPs was evaluated by Helios NanoLab 650 Focused Ion Beam-Scanning Electron Microscope (FIB-SEM) (FEI, Hillsboro, Oregon, USA).

*Encapsulation efficiency and loading content* To evaluate the encapsulation efficiency (EE) and loading content (LC) of insNPs, the unencapsulated insulin was purified from ultrafiltration tube and quantified using the method used by Guo et al.^10^. Briefly, a Agilent 1100 series HPLC system (Agilent, Santa Clara, California, USA) composed of a quaternary pump, an autosampler, a column heater, and a DAD detector was fitted with a C18 column (Zorbax, 3.5 μm, 4.6 mm × 150 mm, Agilent, USA) and eluted with mobile phase (acetonitrile and water with 0.1% of TFA in a gradient ratio from 10/90 to 100/0 for a 10 min run) at a flow rate of 1.0 mL/min. The column temperature was set to 20 °C. EE and LC in percentages were calculated using Eq. (1) and Eq. (2).

1 $$Entrapment\;efficiency \left( \% \right) = \left( {{\raise0.7ex\hbox{${1 - Unencapsulated\;insulin}$} \!\mathord{\left/ {\vphantom {{1 - Unencapsulated\;insulin} {Total\;insulin }}}\right.\kern-0pt} \!\lower0.7ex\hbox{${Total\;insulin }$}}} \right) \times 100\%$$

2 $$Loading\;content \left( \% \right) = \left( {{\raise0.7ex\hbox{${Weight\;of\;insulin}$} \!\mathord{\left/ {\vphantom {{Weight\;of\;insulin} {Weight\;of\;insNPs }}}\right.\kern-0pt} \!\lower0.7ex\hbox{${Weight\;of\;insNPs }$}}} \right) \times 100\%$$

*Reconstitution Ability* All dehydrated insNPs were redissolved in deionized double distilled water to evaluate their reconstitution abilities. The particle sizes, PDI, EE and LC were tested again using the same methods mentioned before.

*In-vitro release behavior* The in-vitro release behaviors of the redissolved insNPs coated with TG-chitosan and MNA-TG-chitosan were tested by dialysis sack method (cutoff molecular weight 100 kDa, Spectra Por Inc.) used by Guo et al.^10^ earlier. Briefly, insNPs were placed in dialysis bag and dialyzed at pH 2.5, pH 6.6, and pH 7.0 incubated at 37 °C with continuous shaking at 200 rpm simulating the pH environments in stomach, duodenum, and upper small intestine, respectively. One mL of the fluid outside the dialysis sack were withdrawn at 30 min, 1 h, 2 h, 3 h, 4 h and 6 h was analyzed for insulin by HPLC method mentioned above, while replenishing the setup with fresh dialysis fluid. The release rate of insulin from insNPs was calculated from the ratio of released as free insulin to the total insulin encapsulated in nanoparticles.The results were compared for unmodified chitosan, TGA-chitosan and MNA-TG-chitosan insNPs.

### In-vitro toxicity, cellular uptake and insulin transport for insNPs

*Cytotoxicity on HepG2, Caco2 and TR146 cells* The MTT test was used to assess the cytotoxicity of insNPs coated with chitosan, TG-chitosan and MNA-TG-chitosan after reconstitution. The HepG2 cells, Caco2 cells and TR146 cells were seeded at a density of 5 × 104, 5 × 104 and 1 × 104 cells/cm^2^ in 96 well plates. insNPs were diluted to various concentration (50 to 1000 μg/mL) in respective culture medium and then were given to the cells. After 8 h incubation, the cells were washed with PBS for three times and refreshed with medium containing 0.5 mg/mL of MTT for another 4 h incubation. The cytotoxicity was evaluated by measuring the enzymatic reduction of yellow tetrazolium MTT to purple formazan at 570 nm using Tecan infinite M200 pro spectrophotometer plate reader (Tecan, Männedorf, Switzerland)^10^.

*Cellular Uptake Efficacy on HepG2 cells* The insNPs’ cellular uptake efficacy was tested by confocal laser scanning microscope and flow cytometry analysis as discussed earlier^10^. The HepG2 cells were seeded at a density of 5 × 10^4^ cells/cm^2^ in Nunc Lab-Tek chamber slide system. Each well of Nunc Lab-Tek chamber slide system was treated with free FITC-insulin, FITC-insNPs coated with chitosan, TG-chitosan and MNA-TG-chitosan at the same concentration of 25 μg/mL and incubated for 4 h. Cells were then fixed with 4% paraformaldehyde and the nuclei of the cells were stained with DAPI. The localization of insulin was observed using an Olympus FV1000 laser scanning/two-photon Confocal Microscope (Olympus, Shinjuku City, Tokyo, Japan)^10^.

*Flow Cytometry on HepG2 Cells* For flow cytometry analysis, HepG 2 cells were seeded at a density of 5 × 10^4^ cells/cm^2^ in 96 well plates. Free FITC-insulin, FITC-insNPs coated with chitosan, TG-chitosan and MNA-TG-chitosan with the concentration of 10 μg/mL were added into the 96-well plate and incubated for 4 h. After 4 h incubation, cells were lifted and washed three times with FBS. 5 × 10^4^ cells per sample were analyzed by BD LSR II flow cytometer (BD, Franklin Lakes, New Jersey, United States)^10^.

*Insulin Transport on TR146 cells* To evaluate permeability, insNPs were tested using Corning^®^ (Transwell pore diameter 0.4 μm, growth area 0.33 cm^2^) inserts with a 24-well plate. In brief^10^, TR146 cells were cultured on the insert for 30 days at a density of 5 × 104 cells/cm^2^, with the medium being changed every two days. Free insulin, insNPs coated with chitosan, TG-chitosan and MNA-TG-chitosan were tested in 24-well plate, respectively. From the receptor portion, 0.1 mL of the sample was taken at 0.5, 1, 2, 3, 4, 6 h for insulin content measurement using the same HPLC method described in previous section. The receptor portion was immediately refilled with fresh PBS in order to maintain the sinking conditions.

*Tight Junction test on TR146 cells* By studying the changes in transepithelial electrical resistance (TEER) values of a monolayer of epithelial cells after the insNP treatment, the opening of tight junctions has been studied. This test was carried out using Corning^®^ (Transwell pore diameter 0.4 μm, growth area 0.33 cm^2^) inserts with a 24-well plate. TR146 cells were cultured on the insert for 30 days at a density of 5 × 10^4^ cells/cm^2^. The cells were incubated with free insulin, insNPs encapsulated with chitosan, TG-chitosan and MNA-TG-chitosan, respectively and the changes in TEER values were measured with a Millicell^®^-Electrical Resistance System at different time intervals within 4 h (Millipore, MA, USA). After incubation, the test sample was removed and TEER values were monitored for another 20 h.

The status of tight junction between the TR146 cells after the incubation with NPs was visualized by the immunofluorescent staining of ZO-1 protein^10^. The TR146 cells were seeded at a density of 5 × 10^4^ cells/mL in Nunc Lab-Tek chamber slide system. The TR 146 cell monolayer was incubated with free insulin, insNPs coated with chitosan, TG-chitosan and MNA-TG-chitosan for 4 h, and then the cells were fixed with 4% paraformaldehyde. The cells were then permeabilized with 0.1% Triton X-100, and blocked with 5% goat serum. Subsequently, cells were treated with ZO-1 monoclonal antibody (Thermo Fisher, NY, USA) followed by Goat anti-Mouse IgG (H + L) Cross-Adsorbed Secondary Antibody, FITC (Thermo Fisher, NY, USA). The stained cells were washed three times with PBS, mounted on slides and visualized using Olympus FV1000 laser scanning/two-photon Confocal Microscope (Olympus, Shinjuku City, Tokyo, Japan).

### Statistical analysis

All experiments were performed in triplicates and values are expressed as Mean ± standard deviation (SD). Comparisons among all groups were evaluated using One-way ANOVA or t-test by IBM SPSS Statistics 26 for Mac (IBM, Endicott, New York, USA), and p < 0.05 was considered to be statistically significant.

### Ethics approval

The biosafety protocols were approved via Application Number B17-0247 to the Biosafety Committee of the University of British Columbia.

## Conclusion

In order to improve the cell tight junction opening capability of chitosan to enable non-parenteral delivery, a new material MNA-TG-chitosan was synthesized by activating the thiol group on TG-chitosan with MNA dimers. The newly synthesized material was characterized to replace > 60% of thiols with disulfide linkages stabilizing the TG-chitosan in acidic environments permitting it to be used for oral delivery. On using MNA-TG-chitosan in spray drying encapsulation of insulin into insNPs, particle size of optimized chitosan insNPs reduced from 318 to 277 nm for MNA-TG-chitosan. insNPs encapsulated with MNA-TG-chitosan exhibited significantly higher cellular uptake by HepG2 liver cells and transepithelial transport through TR146 buccal cell monolayers. The faster action of the MNA-TG-chitosan was primarily attributed to the opening of cell tight junctions demonstrated by reversible reduction in transepithelial resistance of TR146 monolayers. MNA-TG-chitosan showed no significant effect on cell viability confirming that their toxicity is similar to chitosan and TG-chitosan which are regulatory approved for drug delivery applications. This research will allow the use of MNA-TG-chitosan and its’ further derivatives as functional thiomers in the search of appropriate solutions to replace the current subcutaneous injection for peptide delivery.

## Data Availability

Although all related data are provided in the manuscript, any other pertinent information is available on request from the corresponding author (A.P.).
